# Clinicopathological Features and Oncological Outcomes of Early and Late Recurrence in Stage III Colorectal Cancer Patients after Adjuvant Oxaliplatin-Based Therapy

**DOI:** 10.1155/2023/2439128

**Published:** 2023-01-06

**Authors:** Yu-Tang Chang, Hsiang-Lin Tsai, Yen-Cheng Chen, Ching-Chun Li, Ching-Wen Huang, Po-Jung Chen, Wei-Chih Su, Tsung-Kun Chang, Yung-Sung Yeh, Tzu-Chieh Yin, Jaw-Yuan Wang

**Affiliations:** ^1^Division of Pediatric Surgery, Department of Surgery, Kaohsiung Medical University Hospital, Kaohsiung Medical University, Kaohsiung 80708, Taiwan; ^2^Department of Surgery, Faculty of Medicine, College of Medicine, Kaohsiung Medical University, Kaohsiung 80708, Taiwan; ^3^Division of Colorectal Surgery, Department of Surgery, Kaohsiung Medical University Hospital, Kaohsiung Medical University, Kaohsiung 80708, Taiwan; ^4^Graduate Institute of Clinical Medicine, College of Medicine, Kaohsiung Medical University, Kaohsiung 80708, Taiwan; ^5^Department of Surgery, Faculty of Post-Baccalaureate Medicine, College of Medicine, Kaohsiung Medical University, Kaohsiung 80708, Taiwan; ^6^Division of Trauma and Surgical Critical Care, Department of Surgery, Kaohsiung Medical University Hospital, Kaohsiung Medical University, Kaohsiung, Taiwan; ^7^Department of Emergency Medicine, Faculty of Post-Baccalaureate Medicine, College of Medicine, Kaohsiung Medical University, Kaohsiung 80708, Taiwan; ^8^Graduate Institute of Injury Prevention and Control, College of Public Health, Taipei Medical University, Taipei 11031, Taiwan; ^9^Division of General and Digestive Surgery, Department of Surgery, Kaohsiung Medical University Hospital, Kaohsiung Medical University, Kaohsiung 80708, Taiwan; ^10^Department of Surgery, Kaohsiung Municipal Tatung Hospital, Kaohsiung Medical University, Kaohsiung 80145, Taiwan; ^11^Graduate Institute of Medicine, College of Medicine, Kaohsiung Medical University, Kaohsiung 80708, Taiwan; ^12^Center for Cancer Research, Kaohsiung Medical University, Kaohsiung 80708, Taiwan; ^13^Pingtung Hospital, Ministry of Health and Welfare, Pingtung 90054, Taiwan

## Abstract

**Aims:**

An adjuvant oxaliplatin-based regimen is the standard of care for patients with stage III colorectal cancer (CRC). Few reports have compared the clinicopathological features and oncological outcomes of such treatment between patients with early (≤1 year) and late recurrence (>1 year).

**Methods:**

Between January 2012 and December 2019, CRC recurred in 128 (24.1%) of 531 patients with consecutive stage III CRC after they received curative resection and an adjuvant oxaliplatin-based regimen. The clinicopathological features and oncological outcomes of the 128 patients were analyzed retrospectively.

**Results:**

The median follow-up period after the first chemotherapy cycle was 35.0 months (range, 7–100.9), and the median recurrence time was 16.1 months. Forty-seven patients (36.7%) had an early recurrence and eighty-one patients (63.3%) had a late recurrence. Compared with patients with late recurrence, those with early recurrence were mostly younger (median: 58 vs. 64 years, *p*=0.009), had less oxaliplatin-based therapy cycles (median: 8 vs. 12 cycles, *p* < 0.001), and had a shorter overall survival time (median: 23.3 vs. 39.7 months, *p* < 0.001). The area under the curve of patient age and chemotherapy cycles for predicting early recurrence was 0.629 and 0.705 (*p*=0.015 and *p* < 0.001), respectively. The receiver operating characteristic curve analysis demonstrated that the cutoff level for patient age was 57 years and the number of chemotherapy cycles was 8. A multivariate analysis revealed that patient age ≤57 years and oxaliplatin-based therapy ≤8 cycles were independent risk factors for early recurrence (odds ratio (OR) = 3.049, *p*=0.022; OR = 4.995, *p*=0.002). These factors were associated with an approximately 77.8% risk of recurrence within 1 year, compared with the 21.5% risk associated with patient age >57 years and oxaliplatin-based therapy >8 cycles (*p* = 0.003).

**Conclusion:**

Patients with early recurrence had poorer survival than those with late recurrence. If >8 cycles of oxaliplatin-based therapy can be administered without disease progression, then patients with stage III CRC would have a lower risk of early recurrence.

## 1. Introduction

Colorectal cancer (CRC) is the third most commonly diagnosed cancer, with an estimated 1.9 million cases and 915,880 deaths reported in 2020 worldwide [1]. On the basis of findings from an 8-yearfollow-up, the ACCENT group reported that 32.9% of patients with stage III CRC had cancer recurrence [2]. Moreover, 82% of patients with stage III CRC experienced recurrence within the first 3 years of cancer diagnosis, and the incidence of recurrence peaked between 1 and 2 years after initial treatment [2, 3].

According to the Ministry of Health and Welfare of Taiwan, CRC has become the most common cancer and the third leading cause of cancer-related death since 2006. In 2018, the CRC incidence was 41.8 per 100,000 individuals in Taiwan, with 14.9 deaths per 100,000 individuals [4]. Furthermore, a large proportion (25%) of patients with CRC in Taiwan have stage III CRC [4]. The overall survival (OS) rate of patients with stage III CRC was 59.9%.

Adjuvant chemotherapy, in which oxaliplatin is combined with a fluoropyrimidine (FOLFOX or CAPOX) is the standard of care for patients with stage III CRC [5]. Even with curative surgery and adjuvant chemotherapy, the overall prognosis of stage III CRC remains unsatisfactory, with a 5-year survival rate of only 69% [4]. Moreover, despite adjuvant chemotherapy, patients with locally advanced CRC had an approximately 26.7% risk of relapse within 5 years [6]. Therefore, risk factors predicting the progress, relapse, and metastasis of CRC after adjuvant chemotherapy must be identified.

Several risk factors for stage III CRC recurrence have been identified, including rectal cancer, preoperative and postoperative serum carcinoembryonic antigen (CEA) level (>5.0 ng/mL), postoperative carbohydrate antigen 19-9 level, infiltrative growth patterns, and >3 metastatic lymph nodes [6–8]. However, whether recurrence time would determine outcomes following an adjuvant oxaliplatin-based regimen in patients with stage III CRC who had undergone radical resection remains unclear. We here compared the clinicopathological features and oncological outcomes of such treatment between patients with early (≤1 year) and late recurrence (>1 year).

## 2. Methods

### 2.1. Patients

**Figure 1:**
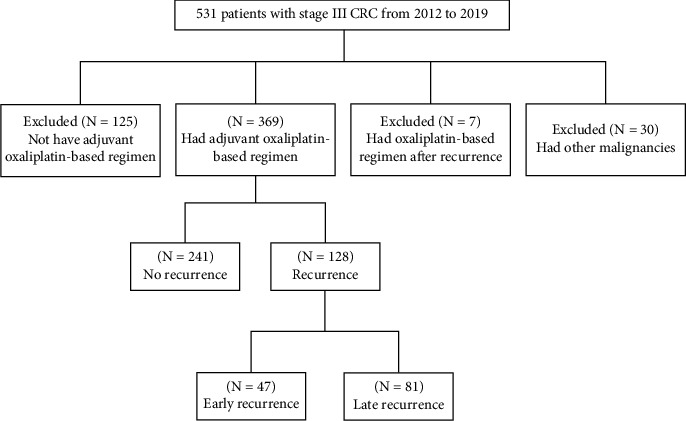
Selection process of 531 patients with stage III colorectal cancer, whose data were collected from the cancer center in our institution (January 1, 2012, to December 31, 2019).

Between January 2012 and December 2019, a total of 531 consecutive patients with histologically confirmed stage III CRC who had undergone surgical treatment at a single institution were analyzed. Of them, 162 patients were excluded because of the following reasons: 125 patients did not receive an adjuvant oxaliplatin-based regimen after surgery; 7 patients started receiving oxaliplatin-based therapy after recurrence; and 30 patients had other malignancies. Finally, 369 patients received the adjuvant oxaliplatin-based regimen after surgery. Of them, 128 patients had a recurrence.  Figure 1 presents the flowchart of patient selection. The present study was approved by the Institutional Review Board of Kaohsiung Medical University Hospital (KMUHIRB-E (I)-20210006). In accordance with the Declaration of Helsinki, this research study was performed in accordance with relevant guidelines, and informed consent was obtained from all the participants.

### 2.2. Chemotherapy

The adjuvant oxaliplatin-based regimen was mFOLFOX and administered as follows: each cycle of FOLFOX comprised oxaliplatin (Eloxatin; 85 mg/m^2^; Sanofi-Aventis, Paris, France) and folinic acid (Covorin; 400 mg/m^2^; Swiss Pharmaceutical, Tainan, Taiwan) on day 1, and a 46-h infusion of 5-FU (2800 mg/m^2^; Nang Kuang Pharmaceutical Co., Ltd, Tainan, Taiwan) was repeatedly administered biweekly for 12 cycles [9, 10].

A 25% dose reduction was according to hematology toxicity such as absolute neutrophil count, platelet count, and nonhematological toxicity including skin symptoms, peripheral neuropathy, and acute laryngopharyngeal dysesthesia if patients were encountered with ≥Grade III adverse events (AEs) [11]. Administration was continued until any of the following criteria for discontinuation were fulfilled [11, 12]:

1. Disease progression was diagnosed clinically or by imaging
2. A Grade III AE occurred again even after dose reduction of chemotherapy regimen
3. A treatment course was delayed for more than 2 cycles of treatment owing to an AE
4. A Grade IV or V AE occurred
5. The patient declined treatment
6. The attending physician judged that continuation of the study was difficult for any other reason

### 2.3. Patient Follow-Up

The clinical stages and pathological features of primary tumors were defined according to the eighth edition of the UICC tumor–node–metastasis staging system [13]. The clinicopathological features analyzed were sex, age, tumor size, tumor location, tumor invasion depth, lymphovascular invasion, perineural invasion, tumor grade, oxaliplatin cycles, and preoperative and postoperative serum CEA levels. Right-sided colon cancers were defined as those located in the cecum, ascending colon, hepatic flexure, and transverse colon, and left-sided cancers were defined as those located in the splenic flexure, descending colon, sigmoid, and rectum.

Treatment responses were assessed using computed tomography, magnetic resonance imaging, or positron emission tomography, and the best responses were recorded. The development of a new instance of local recurrence (tumor growth restricted to the anastomosis or the primary operation region) or distant metastatic lesions (distant metastases or diffuse peritoneal carcinomatosis) during the postoperative surveillance period was defined as postoperative recurrence.

Recurrence within 1 year of the initial treatment with the adjuvant oxaliplatin-based regimen was defined as an early recurrence, and after 1 year was defined as a late recurrence [6]. The progression-free survival (PFS) time was calculated from the date of the first oxaliplatin-based therapy cycle to the date of recurrence. The OS time was calculated from the date of the first oxaliplatin-based therapy cycle. PFS and OS times were analyzed. All patients were followed up until their deaths, their last follow-up date, or May 2021.

### 2.4. Statistical Analysis

Student's *t* test and a chi-square test were used to compare continuous and categorical descriptive variables, respectively, between the groups. The univariate and multivariate logistic regression analyses were used to examine the relationships between clinicopathological features and recurrence. Cumulative PFS and OS rates were calculated using the Kaplan–Meier method, and the log-rank test was used to compare time-to-event distributions. The predictive ability of patient survival was evaluated through receiver operating characteristic (ROC) curve analysis. The area under the curve (AUC) was also calculated. Results were expressed as the mean ± standard deviation or odds ratio (OR) and 95% confidence interval (CI) were appropriate. A *p* value <0.05 indicated statistical significance. All data were analyzed using SPSS (Version 19.0; SPSS, Chicago, IL, USA).

## 3. Results

### 3.1. Descriptive Data

The median follow-up period after the first chemotherapy cycle was 35.0 months (range, 7–100.9). Of the 128 patients, 79 were men (61.7%). The median age was 61.0 years (range, 30–86). Regarding tumor histology, none were well-differentiated, 110 (85.9%) were moderately differentiated, and 18 (14.1%) were poorly differentiated carcinomas. Furthermore, 10 (7.8%) patients were in stage IIIA, 83 (64.8%) in stage IIIB, and 35 (27.3%) in stage IIIC. The median number of cycles of the adjuvant oxaliplatin-based regimen was 12, and 78 patients (60.9%) completed the full 12 chemotherapy cycles. Seventy-nine patients (61.7%) developed distant metastasis and forty-nine patients had local recurrence. During follow-up, 80 patients (62.5%) of 128 patients died. Of the 128 patients, the estimated 5-year OS rate was 35.1% and the median OS time was 48.0 months (95% CI: 37.3–58.8). The clinicopathological characteristics of all 128 patients with stage III CRC with recurrence are listed in Table 1.

### 3.2. Early Recurrence Compared with Late Recurrence

The median recurrence time after the first oxaliplatin-based therapy cycle was 16.1 months. Forty-seven patients (36.7%) had relapse within 12 months (early recurrence) and eighty-one patients (63.3%) had relapse after 12 months (late recurrence) (Table 2). Between the two groups, sex, tumor size, tumor invasion depth, histology, and preoperative and postoperative CEA levels did not significantly differ (all *p* > 0.05). The expression of recurrence did not differ significantly between the patients with local recurrence and those with distant metastasis (*p*=0.449).

**Figure 2:**
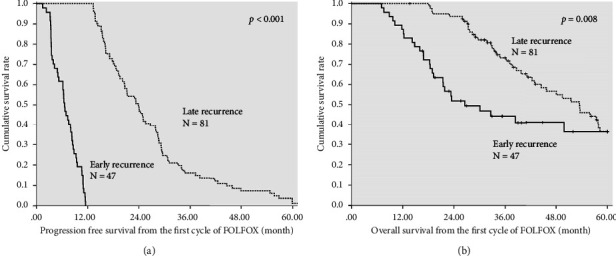
Early versus late recurrence. (a) Cumulative progression-free survival rate from the first chemotherapy cycle and (b) 5-year overall survival rate from the first chemotherapy cycle calculated using the Kaplan–Meier method.

The median PFS time was significantly shorter among the patients with early recurrence than among those with late recurrence (6.6 [95% CI: 5.8–7.4] months vs. 23.9 [95% CI: 20.3–27.4] months, *p* < 0.001; Figure 2(a)). The estimated median 5-year OS time was significantly shorter among the patients with early recurrence than among those with late recurrence 26.5 [95% CI: 13.8–39.3] months vs. 53.4 [95% CI: 43.2–63.6] months, *p*=0.008; Figure 2(b)).

### 3.3. Association between Patient Age and Recurrence

Compared with the patients with late recurrence, those with early recurrence were younger (median 58 years vs. 64 years, *p*=0.009). The predictive ability of patient age for early recurrence was evaluated through the ROC curve and AUC analyses. The AUC of patient age for predicting early recurrence was 0.629 (*p*=0.015). The ROC curve analysis demonstrated that the cutoff level was 57 years. Thus, patients aged >57 and ≤57 years were defined as “older adult patients” and “young patients,” respectively. In the univariate logistic regression analysis, young age was revealed as a predictor for early recurrence (OR = 2.872, 95% CI: 1.330–6.200; *p*=0.007, Table 3).

### 3.4. Association between Cycles of the Adjuvant Oxaliplatin-Based Regimen and Recurrence

Compared with the patients with late recurrence, those with early recurrence had fewer cycles of adjuvant oxaliplatin-based therapy (median 8 cycles vs. 12 cycles, *p* < 0.001). The predictive ability of these cycles for early recurrence was also evaluated through the ROC curve and AUC analyses. The AUC of these chemotherapy cycles for predicting early recurrence was 0.705 (*p* < 0.001). The ROC curve analysis indicated that the cutoff level was eight cycles. Specifically, when the patients received >8 cycles of adjuvant oxaliplatin-based therapy, the analysis result was defined as “positive,” whereas when the patients received ≤8 cycles of adjuvant chemotherapy, the analysis result was defined as “negative.”

Forty patients (31.3%) received ≤8 cycles of adjuvant oxaliplatin-based therapy. Chemotherapy was terminated in 8 of the 40 patients because most of them were intolerant or oxaliplatin toxicity reluctant to further therapy. According to the univariate logistic regression analysis, the patients with a negative result had a 6-fold higher risk of early recurrence than those with a positive result (OR = 5.925, 95% CI: 2.626–13.371; *p* < 0.001, Table 3).

### 3.5. Risk Factors Influencing Early Recurrence

**Figure 3:**
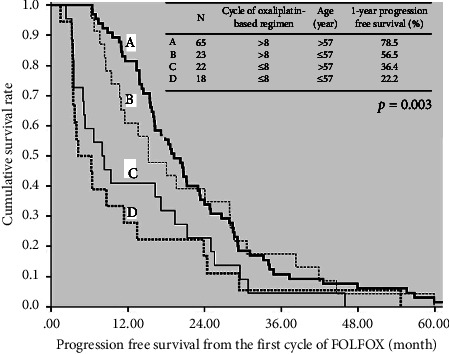
Cumulative progression-free survival of 128 patients calculated using the Kaplan–Meier method. The occurrence of patients aged >57 years with >8 cycles of oxaliplatin-based therapy was correlated with the highest 1-year PFS rate (78.5%), whereas the occurrence of patients aged ≤57 years with with ≤8 cycles of oxaliplatin-based therapy was correlated with the lowest 1-year PFS rate (22.2%) (*p*=0.003).

To identify the independent risk factors for early recurrence in patients with stage III CRC, we used a logistic regression model to perform the univariate and multivariable analyses (Table 3). In the multivariate analysis, early recurrence was significantly correlated with patient age ≤57 years (OR = 3.049, 95% CI: 1.171–7.941; *p*=0.022) and oxaliplatin-based therapy ≤8 cycles (OR = 4.995, 95% CI: 1.806–13.815; *p*=0.002). PFS was further analyzed on the basis of the relationship between patient age and adjuvant chemotherapy cycles (Figure 3). Each patient was classified into one of the four following groups: (a) age >57 years and oxaliplatin-based therapy >8 cycles (*N* = 65); (b) age ≤57 years and oxaliplatin-based therapy >8 cycles (*N* = 23); (c) age >57 years and oxaliplatin-based therapy ≤8 cycles (*N* = 22); and (d) age ≤57 years and oxaliplatin-based therapy ≤8 cycles (*N* = 18). A significant difference in PFS was noted among the four groups (*p*=0.003). The 1-year PFS rate improved significantly to 78.5% in the patients aged >57 years with >8 cycles of oxaliplatin-based therapy. In the patients aged ≤57 years with >8 cycles of chemotherapy, the 1-year PFS rate was 56.5%. In the patients aged >57 years with ≤8 cycles of chemotherapy, the 1-year PFS rate was 36.4%. In the patients aged ≤57 years with ≤8 cycles of chemotherapy, the 1-year PFS rate was 22.2%.

## 4. Discussion

Oxaliplatin-based regimens have recently become the gold standard in postoperative adjuvant chemotherapy for patients with stage III CRC [14]. However, the choice of an appropriate treatment strategy and decision on therapy duration depend on the associated cumulative neurotoxicity. Studies have demonstrated that oxaliplatin-induced neurotoxicity usually presents at the 8th–10th cycle of FOLFOX [15]. The treatment duration can be reduced from 6 (12 cycles) to 3 months (6 cycles) for patients with a low risk of recurrence; this reduction does not compromise effectiveness and may even significantly decrease the risk of cumulative sensitive neuropathy [5]. In the present study, 40 patients received ≤8 cycles of oxaliplatin-based therapy, and 8 (20%) of them developed intolerance to adverse effects and refused continuous therapy. The period of therapy is a critical determinant of how well the therapy aids survival [16].

Recommendations are shown by the findings of a recent pooled analysis of clinical trials that compared 6 versus 3 months of oxaliplatin-based chemotherapy [17, 18]. According to the international, phase 3 trial conducted at 244 centers of The Short Course Oncology Therapy study, 32.5% of patients with high-risk stage II and stage III CRC who received FOLFOX and were considered at a low recurrence risk may be treated effectively and experience less neurotoxicity with 3 months of oxaliplatin-based therapy compared with the standard 6-month regimen [19]. However, the International Duration Evaluation of Adjuvant Chemotherapy collaboration, France, in which 90% of patients received mFOLFOX6, could not establish noninferiority for 3 months of oxaliplatin-based chemotherapy [20]. The results differed depending on the treatment regimen and patient risk group. However, our results revealed the significance number of cycles of treatments in reducing the risk of early recurrence in the adjuvant mFOLFOX6 regimen.

For patients at a high recurrence risk (T4 and N2 subgroups), adjuvant chemotherapy should be offered for 6 months [20]. For patients at a low recurrence risk (T1, T2, or T3 and N1 subgroups), 6 or 3 months of adjuvant chemotherapy may be offered depending on whether a potential reduction in adverse events and no significant difference in PFS were observed with the 3-month regimen [5, 17]. However, neither tumor invasion depth nor lymph node metastasis differed significantly between the early and late recurrence groups in the present study. Pathologic staging is not an effective predictor of early recurrence risk. Tsai et al. recommended at least seven cycles of FOLFOX for favorable PFS and eight cycles for favorable OS [16]. In the present study, 78 patients (60.9%) completed the full 12 cycles of adjuvant oxaliplatin-based therapy; however, 18 (23.1%) of them developed early recurrence. It is difficult to balance between overtreatment (which exposes the patient to unnecessarily high levels of chemotherapy toxins) and undergo treatment (which leaves a high risk of early recurrence unaddressed).

Among populations with recurrence, young patients had a 3-fold higher risk of early recurrence than the older adult patients (OR = 3.049, *p*=0.022). Of the 41 patients aged ≤57 years in the present study, 18 (43.9%) patients received ≤8 cycles of adjuvant oxaliplatin-based chemotherapy. Although these young patients with ≤8 cycles of adjuvant chemotherapy had the worst 1-year PFS rate of 22.2%, they were more likely to receive second-line adjuvant chemotherapy or reoperation than the older patients were. Younger patients with stage III CRC might have a relatively lower risk of early recurrence (1-year PFS rate of 56.5%), if >8 cycles of adjuvant chemotherapy could be administered.

Compared with patients aged <70 years, patients aged >70 years have a higher benefit and safety profile of adjuvant fluorouracil-based chemotherapy than surgery alone [21, 22]. Although only 29 patients were aged ≥70 years (22.7%) in the present study, the 1-year PFS rate improved significantly to 78.5% in the patients aged >57 years with >8 cycles of oxaliplatin-based therapy. In patients with stage III CRC who are older, who have multiple comorbidities, and who are less likely to receive chemotherapy, adjuvant chemotherapy might be associated with a lower death risk [23].

An abnormal preoperative and postoperative serum CEA level (≥5 ng/mL) was a significant independent negative predictive factor for postoperative recurrence [6, 7]. In the present study, neither the preoperative nor the postoperative serum CEA level was a risk factor for early recurrence. However, patients with an abnormal postoperative serum CEA level might have a high risk of postoperative recurrence and mortality. For these patients, >8 cycles of adjuvant oxaliplatin-based therapy could be administered to lower the risk of early recurrence.

Although well-differentiated adenocarcinoma is usually common in CRC, the histological subclassification has a great impact on the prognosis. Imai et al. reported poorly differentiated adenocarcinoma of the CRC had significantly worse PFS and OS than well-to-moderately differentiated adenocarcinoma [24], reflecting that none of the recurrence were well-differentiated in the present study.

The 5-year OS rate of stage III CRC from recurrence was 13.5%, which was similar to the survival rate of 13.8% for the distant-stage disease of CRC according to data from the Surveillance, Epidemiology, and End Results program [25, 26]. In people with unresectable metastatic CRC, recent clinical trials have demonstrated that tailoring treatment to the molecular and pathological features of the tumor improves OS [1]. Therefore, genomic profiling might be necessary to extend survival benefits and reduce drug toxicity. In patients with stage III CRC after adjuvant chemotherapy, the expression of the epidermal growth factor receptor can be used to predict OS and PFS times and postoperative relapse [6, 9].

This study has some limitations. First, this was a retrospective study conducted at a single center. Second, the toxicities of chemotherapy and subsequent lines of therapy were not discussed in the study. Third, various biomarkers for stage III CRC with their possible association with the efficacy of adjuvant chemotherapy were not analyzed. A comparison of heterogeneous populations was not easy because the disease severity and therapeutic strategies between early and late recurrence groups were not completely comparable. Predicting the risk of early recurrence effectively for each patient individually is difficult. In addition, as to “cancer recurrence and metastasis,” it indeed is a very complex progression. Emerging interesting studies show that it should be thought as a bidirectional process that has “self-seeding” in breast cancer, CRC, and nasopharyngeal carcinoma [27, 28]. Moreover, when combined with 5-fluorouracil and oxaliplatin, metformin potentially acts as an adjunctive agent to eliminate CRC *in vitro* and *in vivo* [10]. However, the present study used real-world data and explored the outcomes of patients with stage III CRC with recurrence after curative resection and adjuvant oxaliplatin-based therapy.

## 5. Conclusions

Patients with early recurrence had a poorer OS than those with late recurrence. Young age (≤57 years) and few cycles (≤8 cycles) of an adjuvant oxaliplatin-based regimen are independent risk factors for early recurrence. For patients at increased risk of recurrence from stage III CRC, if >8 cycles of adjuvant oxaliplatin-based therapy could be administered, people of all ages would have a low risk of early recurrence and might have a better OS.

## Figures and Tables

**Table 1:** Clinicopathological features of 128 stage III CRC patients developed recurrence after adjuvant oxaliplatin-based regimen.

Characteristics	*N*	(%)
*Gender*		
Female	49	(38.3)
Male	79	(61.7)
*Age (years)*		
≤57	41	(32.0)
>57	87	(68.0)
*Tumor size (cm)*		
≥5	47	(36.7)
<5	81	(63.3)
*Tumor location*		
Right-sided colon	42	(32.8)
Left-sided colon	86	(67.2)
Pathological staging		
*Depth of tumor invasion*		
T1 + T2 + T3	102	(79.7)
T4	26	(20.3)
*Lymph node metastasis*		
N1	68	(53.1)
N2	60	(46.9)
*Histopathology grade*		
Well differentiated	0	(0)
Moderately differentiated	110	(85.9)
Poorly differentiated	18	(14.1)
*Lymph-vascular invasion*		
No	43	(33.6)
Yes	85	(66.4)
*Perineural invasion*		
No	56	(43.7)
Yes	72	(56.3)
*Preoperative serum CEA level (ng/ml)*		
<5	75	(58.6)
≥5	53	(41.4)
*Postoperative serum CEA level (ng/ml)*		
<5	107	(83.6)
≥5	21	(16.4)
*Cycles of oxaliplatin-based regimen (cycles)*		
≤8	40	(31.2)
>8	88	(68.8)
*Pattern of recurrence/distant metastasis*		
Local recurrence	49	(38.3)
Distal metastasis	79	(61.7)
*Recurrence from first cycle of mFOLFOX*		
Early recurrence	47	(36.7)
Late recurrence	81	(63.3)

CEA: carcinoembryonic antigen.

**Table 2:** Comparison between early recurrence and late recurrence of 128 stage III CRC patients after the adjuvant oxaliplatin-based regimen.

	Early recurrence (*N* = 47)		Late recurrence (*N* = 81)		*p*
	*N*	(%)	*N*	(%)	
*Gender*					0.708
Female	17	(36.2)	32	(39.5)	
Male	30	(63.8)	49	(60.5)	
*Age (years)*					0.006
≤57	22	(46.8)	19	(23.5)	
>57	25	(53.2)	62	(76.5)	
*Tumor size (cm)*					0.922
≥5	17	(36.2)	30	(37.0)	
<5	30	(63.8)	51	(63.0)	
*Location*					0.869
Right-sided colon	15	(31.9)	27	(33.3)	
Left-sided colon	32	(68.1)	54	(66.7)	
*Pathological staging*					
*Depth of tumor invasion*					0.803
T1 + T2 + T3	38	(80.9)	64	(79.0)	
T4	9	(19.1)	17	(21.0)	
*Lymph node metastasis*					0.722
N1	24	(51.1)	44	(54.3)	
N2	23	(48.9)	37	(45.7)	
*Histopathology grade*					0.463
Well differentiated	0	(0)	0	(0)	
Moderately differentiated	39	(83.0)	71	(87.7)	
Poorly differentiated	8	(17.0)	10	(12.3)	
*Lymph-vascular invasion*					0.759
No	15	(31.9)	28	(34.6)	
Yes	32	(68.1)	53	(65.4)	
*Perineural invasion*					0.204
No	24	(51.1)	32	(39.5)	
Yes	23	(48.9)	49	(60.5)	
*Preoperative serum CEA level (ng/ml)*					0.076
<5	23	(48.9)	52	(63.0)	
≥5	24	(51.1)	29	(35.8)	
*Postoperative serum CEA level (ng/ml)*					0.059
<5	35	(74.5)	72	(88.9)	
≥5	12	(25.5)	9	(11.1)	
*Cycles of oxaliplatin-based regimen (cycles)*					<0.001
≤8	26	(55.3)	14	(17.3)	
>8	21	(44.7)	67	(82.7)	
*Pattern of recurrence/distant metastasis*					0.449
Local recurrence	20	(42.6)	29	(35.8)	
Distal metastasis	27	(57.4)	52	(54.2)	
*Patient survival from first cycle of mFOLFOX*					<0.001
Median (month)	23.3		39.7		

CEA: carcinoembryonic antigen.

**Table 3:** Factors influencing early recurrence estimated by univariate and multivariate logistic regression.

		Univariate regression		Multivariate regression	
		OR (95% CI)	*p*	OR (95% CI)	*p*
Gender	Female	0.868 (0.413, 1.825)	0.708	0.589 (0.231, 1.501)	0.267
	Male	1		1	

Age	≤57 years old	2.872 (1.330, 6.200)	0.007	3.049 (1.171, 7.941)	**0.022**
	>57 years old	1		1	

Tumor size (cm)	≥5	0.963 (0.457, 2.032)	0.922	0.771 (0.305, 1.950)	0.583
	<5	1		1	

Tumor location	Left-sided colon	1.067 (0.495, 2.299)	0.869	1.367 (0.468, 3.994)	0.567
	Right-sided colon	1		1	

Depth of tumor invasion	T4	1.122 (0.455, 2.765)	0.803	1.242 (0.249, 6.193)	0.791
	T1 + T2 + T3	1		1	

Lymph node metastasis	N2	0.877 (0.427, 1.802)	0.722	1.207 (0.474, 3.077)	0.740
	N1	1		1	

Histopathology grade	PD	0.687 (0.250, 1.882)	0.465	1.171 (0.462, 2.967)	0.269
	WD + MD	1		1	

Lymph-vascular invasion	Yes	0.887 (0.413, 1.907	0.759	0.936 (0.355, 2.465)	0.893
	No	1		1	
Perineural invasion	Yes	1.598 (0.774, 3.299)	0.205	0.651 (0.262, 1.619)	0.356
	No	1		1	

Prseoperative serum CEA level (ng/mL)	≥5	1.970 (0.927, 4.186)	0.078	1.902 (0.688, 5.255)	0.215
	<5	1		1	

Postoperative serum CEA level (ng/mL)	≥5	2.514 (0.954, 6.627)	0.062	1.267 (0.325, 4.939)	0.733
	<5	1		1	

Cycles of oxaliplatin-based regimen (cycles)	≤8	5.925 (2.626, 13.371)	<0.001	4.995 (1.806, 13.815)	**0.002**
	>8	1		1	

Pattern of recurrence/distant metastasis	Distal metastasis	1.328 (0.637, 2.771)	0.449	1.029 (0.395, 2.679)	0.953
	Local recurrence	1		1	

WD: well-differentiated; MD: moderately differentiated; PD: poorly differentiated; CEA: carcinoembryonic antigen.

## Data Availability

The results reported in the article can be found including, where applicable, hyperlinks to publicly archived datasets analyzed or generated during the study. By data, we mean the minimal dataset that would be necessary to interpret, replicate, and build upon the findings reported in the article.
